# Too little, too much: a 4-year longitudinal study of within-person deviations in propensity for overinvolvement and student well-being

**DOI:** 10.3389/fpsyg.2026.1870777

**Published:** 2026-08-25

**Authors:** Skyler Wyly, Jinyoung Park, Rick H. Hoyle

**Affiliations:** 1 Department of Psychology and Neuroscience, Duke University, Durham, NC, United States; 2 Department of Psychology and Behavioural Sciences, Aarhus University, Aarhus, Denmark

**Keywords:** propensity for overinvolvement, resilience, RI-CLPM, self-regulation, state, trait, university students

## Abstract

University students face mounting pressure to seize every opportunity, yet the long-term costs of this tendency remain poorly understood. This 4-year longitudinal study examined how the propensity for overinvolvement relates to sleep quality and well-being, and whether resilience buffers its effects. A sample of 370 university students (55% White, 68% female) completed annual Fall assessments of propensity for overinvolvement, resilience, sleep quality, and psychological well-being at orientation and across their four undergraduate years. Using a random intercept cross-lagged panel model with latent interactions, our preregistered analyses yielded no evidence of linear effects: propensity for overinvolvement was not associated with poorer sleep or well-being, nor did resilience moderate these relations. In contrast, exploratory analyses of nonlinear within-person effects revealed a consistent pattern: when students deviated from their own typical level of overinvolvement—becoming either more or less overinvolved than usual—they reported poorer sleep quality and lower well-being, and lower resilience, at the subsequent time point. Because these nonlinear effects were exploratory and the preregistered linear and moderation hypotheses were not supported, we interpret them as hypothesis-generating and in need of replication. Consistent with prior work on overinvolvement, these findings suggest that helping students recognize and maintain a stable, personally sustainable level of engagement, rather than continually maximizing involvement, may be a useful focus for student self-monitoring, academic advising, and campus counseling services.

## 1 Introduction

*He who is everywhere is nowhere.* Seneca

College students are overwhelmed and under pressure (Koehler, 2014; Vye et al., 2007). Students face increasing mental health challenges and stress, with concerns about academic performance, pressure to succeed, and post-graduation plans being top stressors, and changes in sleeping habits, new responsibilities, and increased class workload among the leading sources of stress (Beiter et al., 2015; Gardani et al., 2022; Ross et al., 1999). In the face of these academic pressures, students attempting to excel may feel they need to pursue every available opportunity to the point of overinvolvement, leading to stress and burnout (Porru et al., 2021; Robbins, 2022). Meanwhile, students are engaging in high levels of competitiveness and comparisons among peers (Luthar et al., 2020), and the majority of students report being overwhelmed (Cook, 2007). Though involvement is linked to a range of favorable educational outcomes (Astin, 1997), overinvolvement can have diminishing returns and detrimental effects, suggesting moderate involvement is ideal (Koehler, 2014), and a tendency toward overinvolvement can be maladaptive.

### 1.1 Propensity for overinvolvement, sleep quality, and well-being

Astin’s (1999) theory of student involvement describes involvement as the “amount of physical and psychological energy that the student devotes to the academic experience,” exemplified in how much time students invest in studying, student organizations, and faculty-student interactions. In theory, student involvement is a behavioral mechanism directly related to student learning, personal development, and the effectiveness of an educational policy or practice (Astin, 1999). However, excessive involvement, including the impulse to pursue opportunities for further involvement, may become ineffectual and counterproductive. For example, highly involved students tend to have higher retention and graduation rates, but a curvilinear relationship appears for academic achievement, particularly in later years. With increased involvement beyond a moderate level, academic achievement declines (Koehler, 2014). In addition, intensive academic structures (e.g., team-based learning) that require high levels of active involvement have been shown to have double-edged effects, fostering engagement while simultaneously inadvertently exacerbating student anxiety and fear of negative evaluation (Marques et al., 2025).

In the current research, we focus on the propensity for overinvolvement, defined as the dispositional tendency to become overinvolved by taking on too many activities or commitments, which is attributable, in part, to a fear of missing out on opportunities (Hoyle et al., 2021). While conceptually related to constructs such as excessive engagement, perfectionism, and overcommitment—which all capture a general tendency to do too much—the propensity for overinvolvement represents a distinct psychological drive. Notably, one can be high on the propensity for overinvolvement but low on the others. For example, individuals who tend to become overinvolved are not necessarily perfectionistic; in fact, the sheer volume of activities undertaken by someone prone to overinvolvement might work directly against the goal of perfection. Perfectionism is driven by a desire for flawless execution, whereas the propensity for overinvolvement is driven by a desire to maximize opportunities.

Importantly, our construct of interest is the *tendency* toward overinvolvement, which must be distinguished from the reality of actual overinvolvement. Whereas excessive engagement describes a behavioral reality that can arise from external demands or obligations, the propensity for overinvolvement captures the internal, proactive tendency to seek out and reach a similar state. While actual overinvolvement and excessive engagement are largely synonymous, they separate conceptually when evaluating dispositional tendencies. A student might become excessively engaged due to program requirements, other people’s decisions, or external factors beyond their control, despite having no underlying tendency toward overinvolvement.

Furthermore, while people with a high tendency toward overinvolvement may be more likely to overcommit, not all forms of involvement result from commitments. Overcommitment, like excessive engagement, represents a scheduling reality rather than an underlying disposition. The propensity for overinvolvement differs from overcommitment because individuals can be heavily involved in things that they are not obligated to. Overcommitment is typically characterized by an effort-reward imbalance that leads to a disproportionate exertion of effort often associated with burnout, exhaustion, and loss of efficacy (Håkansson et al., 2020; Shoman et al., 2023). Although students with a high tendency toward overinvolvement are highly likely to overcommit, involvement encompasses a broader scope of academic and social participation that does not always entail an effort-reward imbalance.

Fear of missing out has received substantial attention since the early 2000s. It manifests as a maladaptive state associated with excessive smartphone and social media use, decreased well-being, and distracted learning (Przybylski et al., 2013; Rifkin et al., 2025). Fear of missing out is characterized by apprehension and regret for opportunities a person *could have* capitalized on, thereby driving individuals toward overcommitment and excessive engagement in social or professional activities to avoid the anxiety associated with potential loss or exclusion. The tendency to overcommit coupled with a persistent concern about being left out of desirable social activities may leave an individual vulnerable to extending themselves beyond their capabilities.

Overcommitment is linked to poorer outcomes for both sleep quality and well-being. Studies in the workplace have shown that overcommitment and effort-reward imbalances were related to lower sleep quality and sleep disturbances (Kudielka et al., 2004). Many students struggle with poor sleep quality, with 27% describing their sleep quality as poor and 62% meeting the Pittsburgh Sleep Quality Index (PSQI) criteria for poor sleep quality (Becker et al., 2018; Lund et al., 2010). Additionally, physiological monitoring suggests that sleep disturbances such as fragmented sleep, irregular respiration, or heart rate variability are often early indicators of underlying stress (Tuan and Loan, 2025). Viewing time as a finite resource, overinvolvement presents a considerable threat to sleep quality; if a student is highly involved but continues to take on additional commitments, often the only option beyond failing to follow through on those commitments is to sacrifice sleep. In terms of well-being, overcommitment partially accounts for the effect of perfectionism on burnout (Philp et al., 2012), suggesting that maladaptive perfectionism might contribute to the tendency to become overinvolved. In addition to its influence on burnout, overcommitment is associated with poor well-being and poor health outcomes (Avanzi et al., 2022; De Jonge et al., 2000). Similarly, in university students, overcommitment and effort-reward imbalances were associated with burnout (Bassanini et al., 2024). These findings suggest that a propensity for overinvolvement may have similar consequences for students’ sleep quality and well-being.

### 1.2 Resilience as an attenuating factor

Though the tendency to become overinvolved is typically linked to poorer sleep quality and diminished well-being, some people thrive under moderate stress—actively juggling overlapping projects and embracing a fast-paced lifestyle. Our study asks whether these self-imposed, anxiety-provoking environments impair sleep and well-being for some but not for others—particularly those high in resilience. Defined as the capacity to bounce back from adversity (Smith et al., 2008), resilience may help people regulate the strain of daily overcommitment, reducing negative spillover (e.g., poor sleep, lowered well-being) and even leveraging these challenges into personal growth. Stress inoculation theory suggests that moderate stress can serve as “practice,” building coping strategies and psychological resources for future adversity (Meichenbaum, 2017). In this light, we investigate whether the propensity for overinvolvement contributes to a resilience-building pathway, preparing students to manage larger stressors without major impacts on well-being. On the other hand, excessive involvement may lead to overwork, chronic sleep deprivation, and subsequent declines in productivity and well-being (e.g., ten Brummelhuis et al., 2025). Following students over time offers an opportunity to examine whether a tendency to become overinvolved is beneficial, detrimental, or both in terms of well-being.

**Figure 1:**
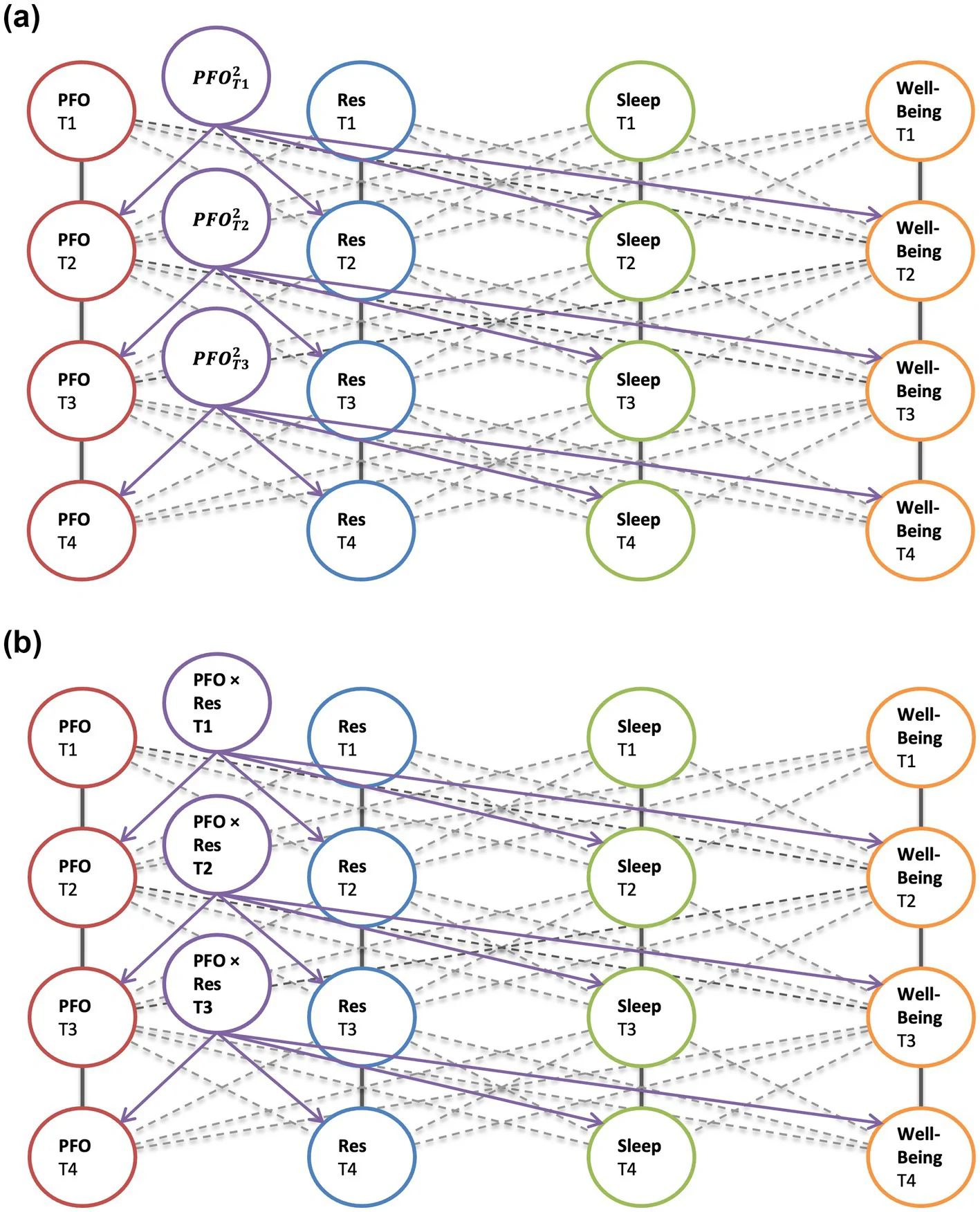
Conceptual model: RI-CLPM with 2-way within-level interactions, and quadratic within-person effects of PFO. *Note*: For both figures, solid grey arrows represent autoregressive effects across time; dashed grey arrows depict cross-lagged effects between all variable pairs; **(a)** solid purple arrows indicate the within-person latent interaction of interest (propensity for overinvolvement × resilience). We also included all pairwise interaction terms at *t*–1 and used them to predict all four variables at *t*. For simplicity, only the interaction between PFO and resilience is depicted in the figure. **(b)** Below is the exploratory model with latent quadratic effect of PFO.

Drawing on 4-year longitudinal study of a student cohort at a highly selective university, we examined the interrelations among propensity for overinvolvement (PFO), resilience, sleep quality, and psychological well-being. Participants completed annual measures of each construct from university entry through their senior year. Using a random intercept cross-lagged panel model with latent interactions, we tested causal pathways linking PFO to sleep quality and well-being, as well as whether trait resilience buffers—or even reverses—the adverse effects of the tendency to become overinvolved (see Figure 1).

Specifically, our hypotheses are:

1. Propensity for overinvolvement will be negatively associated with university students’ sleep quality and overall well-being over time.
2. Resilience will moderate this association, such that the negative effects of propensity for overinvolvement on sleep quality and well-being will be attenuated—or potentially reversed—among students with higher levels of resilience.

In addition, because there are few longitudinal studies on student overinvolvement, we explore the possibility of nonlinear associations between PFO, resilience, sleep quality, and well-being; that is, whether moving either above or below one’s usual level of involvement is maladaptive. Control theory of self-regulation (Carver and Scheier, 1982) offers one rationale: because people monitor their current state against an internal set point and expend regulatory effort to reduce any discrepancy they detect, departures from one’s baseline in either direction, rather than excess alone, may carry costs for subsequent functioning.

## 2 Materials and methods

### 2.1 Participants

The data used for this analysis are from a subsample of participants in the Student Resilience and Well-Being Project (SRWBP; Hoyle et al., 2021), a longitudinal study of students at four residential universities. Data from a single university were used because data collection at this site produced the largest and most complete dataset, making it suitable for our longitudinal structural equation modeling analyses. Participants were full-time, first-year students who were followed from first-year orientation through their fourth year of college. Of the 793 participants who provided data during first-year orientation, 382 agreed to participate in a longitudinal panel of ongoing data collection twice per year across their 4 years at university. A comparison of participants who continued in the study and those who did not on propensity for overinvolvement yielded a significant but small difference, *t*(706) = 2.98, *p* < 0.01, *d* = 0.23, the mean for continuing participants (*M* = 3.82) was higher than the mean for students who did not continue (*M* = 3.63). No significant baseline differences were found between continuing and non-continuing participants on resilience, *t*(712) = −1.34, *p* = 0.180 (continuing *M* = 3.62; non-continuing *M* = 3.54), psychological well-being, *t*(706) = −0.09, *p* = 0.931 (continuing *M* = 4.01; non-continuing *M* = 4.01), or subjective sleep quality, *t*(699) = 0.37, *p* = 0.710 (continuing *M* = 3.01; non-continuing *M* = 3.03). To assess potential attrition bias within the longitudinal panel, we compared the baseline (Year 1) scores of panel participants who completed the final Year 4 assessment to those who dropped out of the study. There were no significant differences between these groups on propensity for overinvolvement, *t*(358) = −0.06, *p* = 0.950, resilience, *t*(367) = 0.34, *p* = 0.732, psychological well-being, *t*(366) = −0.51, *p* = 0.613, or subjective sleep quality, *t*(366) = 1.32, *p* = 0.189. In the longitudinal sample, 68% of the students identified as female and 32% as male. Age ranged from 17 to 21 years (*M* = 18.57). The racial composition of the sample was 16.25% African American/Black, 55.00% White, 15.53% Asian American/Asian, 10.00% Multiracial or Biracial, and 5.53% other. Of the 382 longitudinal panel participants, 370 completed the focal measures; as preregistered, participants missing all four variables were excluded.

### 2.2 Procedure

The study employed a panel design, with data collected twice annually across students’ 4 years at university. Propensity for overinvolvement and psychological well-being were administered annually at the Fall assessment, in October and November of 2015 to 2018. Resilience and sleep quality were measured twice annually, in the Fall assessment and in the Spring assessment, which was administered in February and March; only the annual Fall assessments included all measures for the current study. Compensation for participation included gift cards, clothing, and opportunities to participate in experiences. Efforts at maximizing retention included regular communication with participants and invitations to free meals.

### 2.3 Missing data and power analysis

Two hundred sixty-one participants provided data at all time-points. The total sample sizes were 370, 309, 281, and 281 at the Year 1 to Year 4 assessments, respectively. Students who missed an assessment were encouraged to continue in the study by providing data at subsequent time points. We assumed that the non-responses were missing at random rather than factors related to variables included in our analyses (for details on study logistics, see Hoyle et al., 2021). A Fully Bayesian method (Ibrahim et al., 2005), a Bayesian analog of the full-information maximum likelihood estimator (FIML), is used to account for missing data.

A Bayesian Monte Carlo power analysis was conducted in Mplus (Version 8.7) using the same RI-CLPM specification as the primary analysis, with population values fixed to the posterior medians of the fitted model. Across 50 replications at *N* = 370 over four waves (FBITERATIONS = 10,000 per chain), all 50 replications converged and were retained. Power was high for the quadratic cross-lagged effects of within-person PFO on subsequent outcomes, which were the focal parameters of the present study, exceeding the conventional 0.80 threshold for resilience (power = 0.82), sleep (power = 1.00), and well-being (power = 0.92). Together, these results indicate the present design was well-powered to identify the curvilinear coupling between within-person PFO and subsequent resilience, sleep, and well-being.

### 2.4 Measures

#### 2.4.1 Propensity for overinvolvement

Propensity for overinvolvement, the tendency to become overinvolved and fear of missing out on things, was measured using four items (Hoyle et al., 2021; response scale: 1 = *Not at all true for me* to 5 = *Really true for me*). This measure was designed to concurrently assess both the behavioral tendency to become overinvolved and the underlying psychological apprehension that drives it. The items were: “I feel the need to get involved in lots of different activities,” “I sometimes take on too many commitments,” “I often fear that I will miss out on things,” and “I have a hard time saying ‘no’ to things because I fear I will miss out on something important.” A total score was calculated by computing a mean score. Omega reliability coefficients ranged from 0.77 to 0.81 across the four timepoints. Descriptive statistics and correlations for all variables across four timepoints are reported in the results section, and detailed materials are available on OSF at https://osf.io/z5ymv.

#### 2.4.2 Resilience

To assess individuals’ resilience, or their ability to bounce back and recover from stress, we used the 6-item Brief Resilience Scale (Smith et al., 2008; response scale: 1 = *Strongly disagree* to 5 = *Strongly agree*). This widely used measure was selected because it is designed to capture the capacity to recover from adversity, aligning with our theoretical focus on whether certain students can successfully adapt to the strain of overinvolvement and prevent negative spillover into their health. A sample item is “It does not take me long to recover from a stressful event.” A score was calculated by reverse-scoring the negatively worded items and computing a mean score. Omega reliability coefficients ranged from 0.86 to 0.90.

#### 2.4.3 Sleep quality

Sleep quality was assessed using 15 items from the Pittsburgh Sleep Quality Index (PSQI; Buysse et al., 1989), a widely used and validated self-report measure of sleep disturbances and sleep-related behaviors over the past month. This measure was selected because its assessment of both nocturnal sleep patterns and daytime impairment aligns with our focus on how the physical strain and time constraints of overinvolvement manifest as functional exhaustion. The PSQI includes components such as subjective sleep quality, sleep latency, sleep duration, habitual sleep efficiency, sleep disturbances, use of sleep medication, and daytime dysfunction. Each component is scored on a scale from 0 (no difficulty) to 3 (severe difficulty), with higher scores indicating poorer sleep quality. A global sleep quality score was computed as the mean of the seven component scores; component scoring details are documented in the R code available on OSF.

#### 2.4.4 Psychological well-being

We conceptualize well-being through the eudaimonic perspective, which focuses on meaning, self-realization, and holistic psychological functioning (Ryan and Deci, 2001; Ryff, 1989). Rather than focusing on hedonic aspects such as subjective happiness or positive affect (Diener, 1984), the eudaimonic framework better captures the theorized consequences of overinvolvement. When students become overinvolved, driven by a fear of missing out, it may compromise their autonomy by turning self-determined choices into stressful obligations. As these obligations accumulate, students may lose their sense of environmental mastery, feeling overwhelmed by their schedules rather than in control of them. The resulting exhaustion may leave fewer resources for actual personal growth. To capture this multidimensionality, we used the 6-item Psychological Well-being Index adapted by Konow and Earley (2008) from Ryff and Keyes’s (1995) 18-item theory-based multidimensional model of psychological well-being. This brief index was constructed by taking one highly correlated item from each of the six original psychological well-being dimensions (self-acceptance, positive relations with others, autonomy, environmental mastery, purpose in life, and personal growth; response scale: 1 = *Strongly disagree* to 5 = *Strongly agree*). A sample item is “For me, life has been a continuous process of learning, changing and growth.” A score was calculated by reverse-scoring the negatively worded items and computing a mean score. Omega reliability coefficients ranged from 0.68 to 0.72.

### 2.5 Statistical methods and plan of analysis

A random intercept cross-lagged panel model (RI-CLPM; Hamaker et al., 2015) with latent variables at each timepoint specified with mean scores from scale items for each construct was used to test the hypothesized causal relationship between propensity for overinvolvement, resilience, sleep quality, and psychological well-being. The RI-CLPM allows researchers to distinguish between state changes (within-person) and trait differences (between-person) while examining autoregressive (AR) effects, which refers to the carry-over effect of the previous state of that variable, and cross-lagged (CL) effects, which refer to the effect of other variables at the preceding timepoint. The RI-CLPM addresses several limitations of the cross-lagged panel model (CLPM) that support causal inference. Specifically, Hamaker et al. (2015) note that the traditional CLPM fails to sufficiently account for trait-like, time-invariant stability. Because psychological constructs are frequently characterized by stable individual differences, failing to separate these from temporal variations can confound cross-lagged regression estimates. This confounding “may lead to erroneous conclusions regarding the *presence*, *predominance*, and *sign* of causal influences” (Hamaker et al., 2015, p. 102). By including random intercepts, the RI-CLPM disentangles stable between-person differences from the within-person process. Consequently, the cross-lagged relationships estimated in the RI-CLPM represent actual within-person dynamics, providing a more rigorous foundation for interpreting reciprocal causal influences over time. The study’s design and analysis were preregistered and can be accessed at https://osf.io/6jm3t; research materials and analysis code have been made publicly available on OSF and can be accessed at https://osf.io/z5ymv.

At each wave, these variables are modeled as latent constructs. The model includes:

1. Autoregressive (AR) effects for each variable across time [e.g., ${\mathrm{PFO}}_{\left(t-1\right)}$ → ${\mathrm{PFO}}_{\left(t\right)}$ ]
2. Cross-lagged (CL) effects between all variables [e.g., ${\mathrm{PFO}}_{\left(t-1\right)}$ → ${\text{Sleep}}_{\left(t\right)}$ ; ${\mathrm{Res}}_{\left(t-1\right)}$ → ${\text{Sleep}}_{\left(t\right)}$ ].
3. We will first attempt to estimate within-person latent interactions for all pairwise combinations among the four variables—propensity for overinvolvement (PFO), resilience, sleep quality, and psychological well-being (WB)—to examine whether the relationship between any two variables is moderated by time-specific fluctuations in a third variable. These models will include all two-way within-person interaction terms (e.g., PFO × Res, PFO × Sleep, Res × WB, etc.) at time *t*−1 predicting outcomes at time *t*.

However, if the full interaction model fails to converge due to model complexity or computational limitations, we will proceed with a reduced model that includes only the within-person latent interactions between PFO and Resilience—the primary focus of our hypotheses. Specifically, we will test whether PFO_(*t*−1)_ × Res_(*t*−1)_ predicts Sleep_(*t*)_ and Well-Being_(t)_, capturing the potential moderating role of resilience in the relationship between overinvolvement and downstream outcomes.

As an exploratory analysis, we will examine whether the association between PFO and the outcomes is nonlinear, such that both under- and over-involvement—that is, deviations or fluctuations from one’s usual level of involvement—may be maladaptive.

We assume partial stationarity, constraining all autoregressive and cross-lagged paths, as well as interaction effects, to be time-invariant, while allowing intercepts to vary across time for flexibility.

The specification is as follows. For each outcome variable index d∈V = {PFO, Res, Sleep, WB}, individual *i*, and time *t* = 2, 3, 4:

Model 1: full interaction model


$$\begin{array}{l} Y_{d,i,t}^{\left(W\right)}=\alpha_{d,t}+\zeta_{d,i}+\sum_{a\in V}\delta_{d,a}Y_{a,i,t-1}^{\left(W\right)}\\ +\sum_{a<d\in V}\delta_{d,a,b}Y_{a,i,t-1}^{\left(W\right)}Y_{b,i,t-1}^{\left(W\right)}+\varepsilon_{d,i,t} \end{array}$$

The outcome variable, $Y_{d,i,t}^{\left(W\right)}$ , for each d∈V , represents person-mean–centered latent variables derived from the random-intercept cross-lagged panel decomposition, and a<d∈V denotes unique combination of elements in *V*. The reduced models have $\delta_{d,a,b}=0$ for all (d,a,b) tuples other than (Sleep,PFO,Res) and (WB,PFO,Res).

Model 2: PFO quadratic model


$$\begin{array}{l} Y_{d,i,t}^{\left(W\right)}=\alpha_{d,t}+\zeta_{d,i}+\sum_{a\in V}\delta_{d,a}Y_{a,i,t-1}^{\left(W\right)}\\ +\gamma_{d,\mathrm{PFO}}{\left\{Y_{\mathrm{PFO},i,t-1}^{\left(W\right)}\right\}}^{2}+\varepsilon_{d,i,t} \end{array}$$

where:

$Y_{d,i,t}^{\left(W\right)}$ = person-mean-centered latent outcome variable *d* for person *i* at time *t*

$\alpha_{d,t}$ = time-specific intercept for outcome variable *d*

$\zeta_{d,i}$ = random intercept for person *i* for outcome variable *d*

$\delta_{d,a}$ = linear within-person lagged effect of latent outcome *a* on latent outcome *d*

$\delta_{d,a,b}$ = lagged interaction effect between latent outcomes *a* and *b* on latent outcome *d*

$\gamma_{d,\mathrm{PFO}}$ = quadratic within-person effect of PFO on latent outcome *d*

${\left\{Y_{\mathrm{PFO},i,t-1}^{\left(W\right)}\right\}}^{2}$ = squared within-person deviation of PFO at time *t*−1, capturing the magnitude of departures from a person’s typical level of overinvolvement, regardless of direction

$\varepsilon_{d,i,t}$ = residual error term for individual *i* at time *t*, representing unexplained within-person variation of variable *d*

We will use a Bayesian estimation framework implemented in Mplus 8.7 using Markov Chain Monte Carlo (MCMC) methods via the Gibbs sampler. Non-informative uniform priors will be used. Model convergence will be assessed using the Potential Scale Reduction Factor (PSR), with values <1.1 indicating acceptable convergence. Bayesian estimation provides advantages in modeling latent interactions, handling missing data, and producing stable estimates even in complex models (Asparouhov and Muthén, 2010, 2021).

The means, standard deviations, bivariate correlations of the measurements at each time point, and ICC will be calculated and reported. Model convergence will be assessed using the Proportional Scale Reduction factor, with values below 1.1 indicating acceptable convergence. Individual parameter estimates will be tested for statistical significance using two-sided tests at *α* = 0.05.

For confirmatory factor analysis on the propensity for overinvolvement measure, we will use the conventional criteria to determine the model fit: a Comparative Fit Index (CFI; Bentler, 1990) and Tucker–Lewis Index (TLI; Tucker and Lewis, 1973) of 0.90 or higher (with 0.95 or higher considered good), a Root Mean Square Error of Approximation (RMSEA; Steiger, 1990) of 0.08 or lower (with 0.06 or lower considered good), and a Standardized Root Mean Square Residual (SRMR) of 0.08 or lower (with 0.05 or lower considered good).

Because the latent-interaction and latent-quadratic terms were specified as products of latent variables (XWITH) under TYPE = RANDOM, Mplus does not provide a posterior predictive *p*-value, DIC, or conventional SEM fit indices for the moderation and quadratic models; these indices are available only for the linear model (Hypothesis 1), for which we report the posterior predictive *p*-value. For all models we report the number of free parameters, the number of MCMC chains and iterations, and the achieved Potential Scale Reduction (PSR). For each focal effect we report the posterior median, posterior SD, 95% credible interval, and posterior *p*-value. Convergence diagnostics and fit information for all three models, including the number of free parameters, MCMC chains and iterations, the final PSR, and the posterior predictive *p*-value (available for the linear model only), are summarized in Supplementary Table 1.

## 3 Results

A single-factor confirmatory factor model of the set of items developed to assess the propensity for overinvolvement (see Supplementary Figure 1) was estimated at each timepoint. Measure reliability coefficients and model fit indices for each timepoint are available in Supplementary Table 4. Model fit failed to reach cutoffs for acceptable fit at all timepoints, likely reflecting the multidimensional nature of the construct (see Supplementary Table 5). However, when modeled as two factors, the subscales were strongly correlated across all waves (*r*s ranging from 0.57 to 0.73; see Supplementary Table 5). Given this substantial overlap and the measure exhibiting internal consistency (*ω* = 0.77 to 0.81), mean scores were utilized as planned to maintain consistency with our preregistered analysis and reduce model complexity.

**Figure 2:**
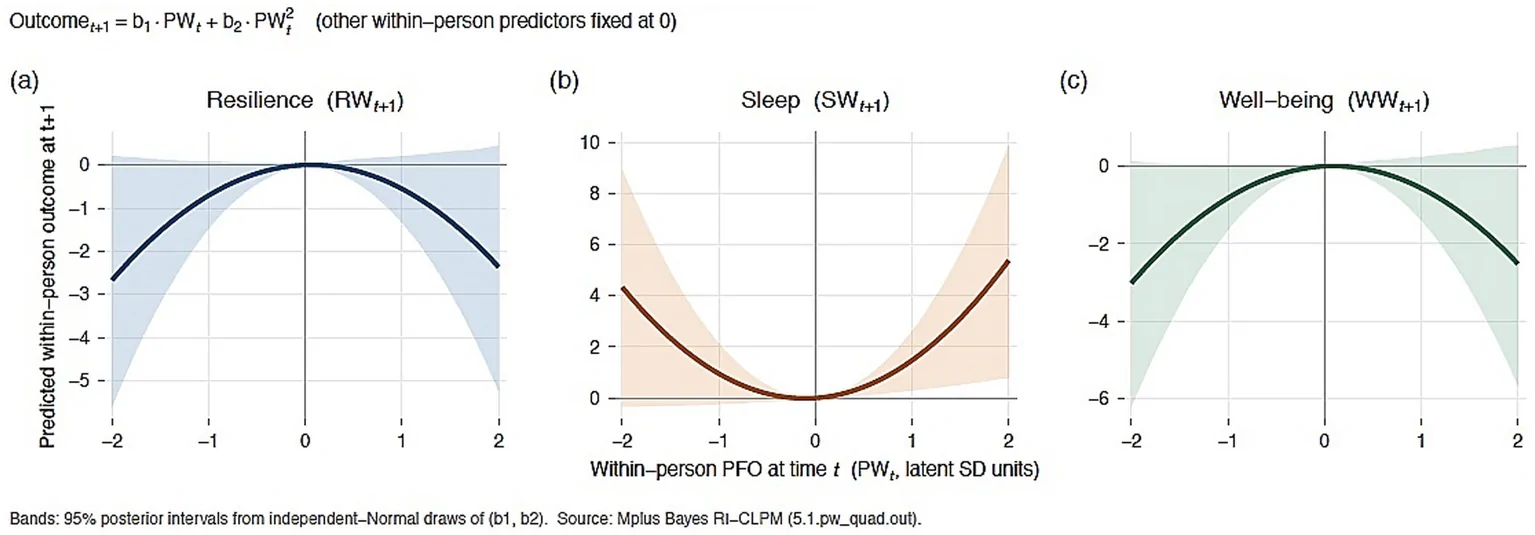
Quadratic within-person effects of PFO on **(a)** resilience, **(b)** sleep quality, and **(c)** well-being. *Note*. Curves depict the estimated within-person quadratic effect of PFO at time *t* on the outcome at time *t* + 1. The *x*-axis represents deviations from an individual’s usual (person-specific) level of PFO (within-person latent factor), and the *y*-axis represents predicted deviations in the subsequent outcome (within-person latent units). Solid lines reflect posterior median estimates from Bayesian estimation. Shaded bands represent approximate 95% credible intervals computed by combining the lower and upper bounds of the linear and quadratic coefficients.

The means, standard deviations, and bivariate correlations of the measurements at each time point are summarized in Table 1. The ICCs (using the *psych* package; Revelle, 2026) for propensity for overinvolvement, resilience, sleep quality, and psychological well-being were 0.42, 95% CI [0.36, 0.47], 0.52, 95% CI [0.47, 0.57], 0.37, 95% CI [0.31, 0.42], and 0.50, 95% CI [0.45, 0.56], respectively, indicating that the within-subject variation of each measure over time is moderately stable compared to the between-individual variability. The main results are summarized in Figure 2, Table 2.

**Table 1:** Descriptive statistics and correlations of propensity for overinvolvement, resilience, sleep quality, and well-being across four time points.

Variable	*M*	*SD*	1	2	3	4	5	6	7	8	9	10	11	12	13	14	15
1. P1	3.82	0.81															
2. P2	3.56	0.90	0.43^**^														
3. P3	3.42	0.96	0.40^**^	0.55^**^													
4. P4	3.27	0.99	0.32^**^	0.48^**^	0.63^**^												
5. R1	3.62	0.78	0.02	0.02	0.00	−0.01											
6. R2	3.50	0.80	−0.08	−0.10	−0.04	0.03	0.51^**^										
7. R3	3.62	0.81	0.00	0.03	−0.06	−0.05	0.46^**^	0.67^**^									
8. R4	3.58	0.86	−0.03	−0.03	−0.08	−0.06	0.39^**^	0.59^**^	0.67^**^								
9. S1	1.05	0.42	0.06	0.03	0.13^*^	0.03	−0.19^**^	−0.20^**^	−0.14^*^	−0.17^**^							
10. S2	0.90	0.39	0.06	0.14^*^	0.14^*^	0.01	0.03	−0.16^**^	−0.13^*^	−0.17^**^	0.37^**^						
11. S3	0.84	0.40	−0.01	0.00	0.08	0.02	−0.07	−0.20^**^	−0.22^**^	−0.21^**^	0.40^**^	0.41^**^					
12. S4	0.83	0.46	0.08	0.08	0.16^**^	0.15^*^	−0.09	−0.15^*^	−0.24^**^	−0.24^**^	0.43^**^	0.36^**^	0.46^**^				
13. W1	4.01	0.59	0.05	0.02	−0.08	−0.02	0.44^**^	0.36^**^	0.39^**^	0.33^**^	−0.18^**^	−0.11	−0.23^**^	−0.13^*^			
14. W2	3.78	0.63	−0.01	−0.01	−0.03	0.01	0.23^**^	0.52^**^	0.43^**^	0.38^**^	−0.14^*^	−0.19^**^	−0.31^**^	−0.17^**^	0.46^**^		
15. W3	3.84	0.65	0.05	0.06	−0.03	−0.01	0.28^**^	0.38^**^	0.53^**^	0.42^**^	−0.17^**^	−0.05	−0.29^**^	−0.26^**^	0.55^**^	0.68^**^	
16. W4	3.85	0.64	−0.04	−0.04	−0.11	−0.06	0.18^**^	0.33^**^	0.38^**^	0.50^**^	−0.14^*^	−0.18^**^	−0.31^**^	−0.28^**^	0.38^**^	0.54^**^	0.62^**^

*M* and *SD* represent mean and standard deviation, respectively. P, propensity for overinvolvement; R, resilience; S, sleep quality (higher scores indicate poorer sleep quality); W, psychological well-being; numbers 1–4 denote measurement time points (Years 1–4). ^*^Indicates *p* < 0.05. ^**^indicates *p* < 0.01.

**Table 2:** Quadratic within-person effects of propensity for overinvolvement on resilience, sleep quality, and well-being.

Outcomes	Predictors	Estimate	*SD*	*p*-value	95% CI
PFO					
	PW_(*t*−1)_	0.56	0.16	0.004	[0.23, 0.85]
	PW^2^_(*t*−1)_	−0.05	0.19	0.758	[−0.42, 0.36]
	RW_(*t*−1)_	0.09	0.08	0.236	[−0.07, 0.25]
	SW_(*t*−1)_	−0.04	0.16	0.806	[−0.38, 0.26]
	WW_(*t*−1)_	0.03	0.13	0.788	[−0.22, 0.31]
Resilience					
	RW_(*t*−1)_	0.65	0.16	0.002	[0.29, 0.92]
	PW_(*t*−1)_	0.08	0.18	0.654	[−0.28, 0.43]
	SW_(*t*−1)_	0.17	0.22	0.402	[−0.23, 0.64]
	WW_(*t*−1)_	0.01	0.17	0.920	[−0.25, 0.44]
	PW^2^_(*t*−1)_	−0.63	0.36	0.010	[−1.52, −0.16]
Sleep quality					
	SW_(*t*−1)_	−0.28	0.13	0.028	[−0.53, −0.03]
	PW_(*t*−1)_	0.26	0.17	0.070	[−0.03, 0.63]
	RW_(*t*−1)_	0.01	0.10	0.904	[−0.18, 0.20]
	WW_(*t*−1)_	−0.12	0.13	0.260	[−0.42, 0.10]
	PW^2^_(*t*-1)_	1.22	0.58	< 0.001	[0.69, 2.90]
Well−being					
	WW_(*t*-1)_	0.51	0.18	0.004	[0.19, 0.92]
	PW_(*t*−1)_	0.13	0.17	0.404	[−0.17, 0.48]
	RW_(*t*-1)_	−0.03	0.11	0.774	[−0.22, 0.21]
	SW_(*t*−1)_	0.33	0.18	0.034	[0.03, 0.71]
	PW^2^_(*t*−1)_	−0.69	0.39	0.002	[−1.74, −0.27]

Within-person effects of PFO (PW) and quadratic PFO (PW^2^) on resilience (RW), sleep quality (SW), and well-being (WW); predictors are time lagged at *t*−1. Estimates represent the posterior medians of the unstandardized path coefficients, standard deviations (*SD*); statistical significance is indicated with two-tailed *p*-values and 95% credible intervals (CIs).

### 3.1 Hypothesis 1

The time-varying outcome model did not support our hypothesis. Changes in propensity for overinvolvement over time did not meaningfully predict later increases or decreases in sleep quality or psychological well-being [for sleep, *b* = 0.04, posterior SD = 0.06, 95% credible interval (−0.07, 0.15), posterior *p* = 0.51, for well-being *b* = 0.01, posterior SD = 0.07, 95% credible interval (−0.12, 0.16), posterior *p* = 0.91]. See Supplementary Table 2.

### 3.2 Hypothesis 2

The interaction model did not support our hypothesis. The interaction effect between PFO and resilience at the previous time point did not meaningfully predict the changes in sleep or well-being in the subsequent time point, [for sleep, *b* = 0.00, posterior SD = 0.08, 95% credible interval (−0.16, 0.15), posterior *p* = 0.98, for well-being *b* = −0.09, posterior SD = 0.10, 95% credible interval (−0.32, 0.10), posterior *p* = 0.32]. See Supplementary Table 3.

### 3.3 Exploratory non-linear effect

After confirming that our hypotheses were not supported, we proceeded to exploratory analyses examining the potential quadratic effects of PFO in the previous time point on all four variables: PFO, resilience, sleep quality, and psychological well-being at their subsequent time point. The quadratic model included 102 free parameters and was estimated with two MCMC chains and 200,000 iterations; convergence was satisfactory, with a final PSR of approximately 1.01 (well below the 1.1 criterion). The near-zero correlations between propensity for overinvolvement and both sleep and well-being (see Table 1) further warranted exploration of possible nonlinear relationships. The quadratic effect reflects within-person nonlinear effects, capturing the extent to which deviations from one’s typical levels of PFO—both above and below their personal means—are associated with subsequent outcomes.

#### 3.3.1 Propensity for overinvolvement

No predictors meaningfully explained subsequent PFO, except for its autoregressive effect [*b* = 0.56, posterior *SD* = 0.16, 95% credible interval (0.23, 0.85), posterior *p* = 0.004]. PFO at the previous time point significantly predicted PFO at the subsequent time point, indicating temporal stability.

#### 3.3.2 Resilience

Resilience showed a meaningful positive autoregressive effect [*b* = 0.66, posterior *SD* = 0.16, 95% credible interval (0.29, 0.93), posterior *p* = 0.002]. In addition, the quadratic effect of PFO ( ${\mathrm{PFO}}_{t-1}^{2}$ ) showed meaningful association with resilience at the subsequent time point [*b* = −0.63, posterior *SD* = 0.36, 95% credible interval (−1.52, −0.16), posterior *p* = 0.01]. This finding suggests that departures from one’s usual level of PFO in either direction (i.e., higher or lower than typical) were associated with lower resilience at the subsequent time point. None of the other cross-lag effects ( ${\mathrm{PFO}}_{t-1},{\text{Sleep}}_{t-1},{\mathrm{WB}}_{t-1}$ ) meaningfully predicted resilience.

#### 3.3.3 Sleep quality

A similar pattern emerged for sleep quality. There was a meaningful negative autoregressive effect of sleep [*b* = −0.28, posterior *SD* = 0.13, 95% credible interval (−0.53, −0.03), posterior *p* = 0.028], indicating that higher levels of poor sleep at one time point were associated with modest improvements at the subsequent time point. In addition, the quadratic effect of PFO ( ${\mathrm{PFO}}_{t-1}^{2}$ ) had a meaningful association with later poor sleep [*b* = 1.22, posterior *SD* = 0.58, 95% credible interval (0.69, 2.90), posterior *p* < 0.001]. This suggests that deviations from one’s usual level of PFO in either direction were associated with worse sleep at the next time point. No other cross-lag effects were meaningful.

#### 3.3.4 Well-being

Well-being showed a meaningful positive autoregressive effect, indicating temporal carryover [*b* = 0.51, posterior *SD* = 0.18, 95% credible interval (0.20, 0.92), posterior *p* = 0.004]. Poor sleep showed meaningful positive cross-lag effect, where increases in poor sleep at the previous time point were associated with greater well-being at the subsequent time point. Again, PFO showed a meaningful quadratic association with well-being [*b* = −0.70, posterior *SD* = 0.39, 95% credible interval (−1.74, −0.27), posterior *p* = 0.002]; deviations from one’s typical level of PFO were associated with a decrease in well-being at the next time point.

To confirm that the curvilinear effects were not an artifact of omitting the resilience interaction, we re-estimated the quadratic model with the within-person PFO × Resilience interaction added simultaneously (Supplementary Table 6). The model converged (final PSR = 1.09), and all three quadratic effects of PFO remained credibly different from zero and in the same direction [resilience, *b* = −1.40, 95% CI (−5.04, −0.27); sleep, *b* = 1.87, (0.81, 5.54); well-being, *b* = −0.90, (−3.54, −0.10)], while the interaction remained non-significant. The curvilinear pattern is therefore robust to inclusion of the moderation term, though the wider credible intervals reflect the added collinearity of jointly estimating both second-order terms.

## 4 Discussion

Before interpreting these findings, we emphasize the distinction between our confirmatory and exploratory results. Our preregistered hypotheses—that propensity for overinvolvement would be linearly associated with poorer sleep and well-being, and that resilience would moderate these associations—were not supported. The nonlinear within-person pattern emerged from exploratory analyses conducted after the preregistered tests were null. We therefore present the curvilinear findings as hypothesis-generating, requiring replication in independent samples before strong conclusions about the costs of being ‘too much’ or ‘too little’ involved are warranted.

In this study, we examined the interactive relationships between a propensity for overinvolvement, resilience, sleep quality, and psychological well-being across 4 years with university students. With the use of an RI-CLPM, we are able to examine within-person state-like changes and between-person trait-like individual differences separately. In this way, we account for how changes in propensity for overinvolvement, resilience, sleep quality, and well-being affect their own trajectory and their effect on other variables at later timepoints. Our focus was on understanding the impact of propensity for overinvolvement on sleep quality and well-being, and the interactive effects of resilience in this relationship. One of the strengths of using an RI-CLPM is its ability to comprehensively examine bidirectional relationships between each variable over time. The inclusion of interaction effects allowed us to examine the interactive properties of resilience in potentially attenuating the consequences of a propensity for overinvolvement on students’ sleep quality and well-being.

We did not find evidence supporting our preregistered predictions that a propensity for overinvolvement would be negatively associated with university students’ sleep quality and overall well-being over time, or that resilience would moderate this association. These results parallel those of a cross-sectional survey of Australian university students, where effort-reward imbalances were related to burnout and withdrawal intentions, but student resilience did not moderate these effects (Williams et al., 2018).

Although resilience did not moderate the effects of overinvolvement, this null result merits interpretation rather than dismissal. The Brief Resilience Scale assesses the trait capacity to recover from stress, which may not capture the moment-to-moment regulatory processes that would buffer the acute strain of becoming overinvolved; the scale’s strong internal consistency (*ω* = 0.86–0.90) makes simple unreliability an unlikely explanation. The annual assessment interval may also be too coarse to detect buffering that operates over days or weeks. In addition, the restricted range of resilience in a highly selective sample may have limited statistical power to detect an interaction. Notably, resilience was not inert in our data: deviations from a student’s typical level of overinvolvement predicted lower subsequent resilience [*b* = −0.63, 95% CI (−1.52, −0.16)]. This suggests that, rather than serving as a buffer, resilience may itself be eroded by instability in overinvolvement, which represents a pathway worth examining directly in future work.

Across outcomes, exploratory analyses revealed a consistent pattern suggesting that departures from individuals’ typical levels of propensity for overinvolvement—whether higher or lower than usual—were associated with poorer subsequent functioning. Specifically, nonlinear effects of PFO predicted subsequent worsening in resilience, sleep quality, and well-being, indicating that both excessive and unusually low engagement may undermine well-being and health over time. Rather than supporting a simple linear “more is better” or “less is better” model, the results point to the potential importance of balance and stability in overinvolvement-related processes (e.g., Carver and Scheier, 1982). Although these analyses were exploratory and require replication, they highlight nonlinear dynamics as a promising direction for future research on overinvolvement and well-being.

Note that the present study examines the propensity for overinvolvement—a disposition or trait that may lead one to become overinvolved—not actual overinvolvement, which was not measured in this cohort. Several studies have examined student involvement levels by measuring hours involved in cocurricular activities and number of extracurricular activities (Koehler, 2014; Berry et al., 2024). Importantly, even with the same workload, people react differently. For example, early work in the stress literature used an events-centered approach which later shifted to measuring perceived stress (Lazarus, 1990). Just as only the person can say whether they are stressed or not, overinvolvement similarly goes beyond pure levels of involvement and should consider the student’s individual threshold for when involvement becomes perceived as overwhelming.

Future longitudinal work could examine the effects of propensity for overinvolvement on stress and examine patterns in propensity for overinvolvement trajectories using latent growth curve models. With a continued propensity for overinvolvement, stressors may accumulate over time to reach an overwhelming level, particularly in academic contexts (Bewick et al., 2010). An alternative approach may be to examine overinvolvement using an index of overinvolvement based on extracurricular and cocurricular activities, both in how a propensity for overinvolvement relates to behavioral manifestations of overinvolvement and the consequences of overinvolvement. Additionally, to avoid biases stemming from self-report approaches, future research could incorporate wearable biosensor technology to capture objective physiological measures of stress and sleep (Norful et al., 2025).

These findings should be considered in context, as participants were from a highly selective university, which may limit generalizability. Additional research with participants from varying socioeconomic and demographic backgrounds would be beneficial. In particular, students from lower income backgrounds are more likely to work while attending college and face additional pressures that may be related to a higher propensity for overinvolvement (Carnevale and Smith, 2018; Davis, 2023; Hosken et al., 2013; Wladis et al., 2024). Qualitative work with underrepresented high school students indicates the pressures of college readiness come at a steep price, leaving these students overwhelmed, scared, and increasingly stressed (Martinez et al., 2020). Overcommitment in a longitudinal occupational health study led to reduced work ability (Håkansson et al., 2020), with findings suggesting potential gender differences in the effects of overinvolvement. Additionally, propensity for overinvolvement has not yet been explored in professional contexts, and has strong implications for burnout, work-life balance, and sustained work performance in organizational settings (Duxbury et al., 2008, 2018).

There are also time and measurement limitations in this study. While the study was designed to observe long-term changes over time, 1-year intervals may be too long to fully capture the impact of state changes in propensity for overinvolvement, self-regulation, resilience, and psychological well-being. Patterns in propensity for overinvolvement may vary across the year, and in the context of an academic year may look different depending on when they are measured during the academic year (Spring, Fall) and during a semester (beginning, middle, end) (Grimm et al., 2012; Pitt et al., 2018). Lastly, while baseline analyses suggest attrition was not systematically linked to our primary health and well-being outcomes, the decreasing analytic sample over the 4 years remains a limitation common to longitudinal research.

The inclusion of a measure for propensity for overinvolvement in this cohort of students was motivated by university administrators recognizing a pattern of overinvolvement in the students they support, and a drive to intervene to mitigate the consequences of this tendency to overreach. This study provides a largescale, exploratory examination of how overinvolvement tendencies may relate to health outcomes, and provides preliminary insights into how stability in such tendencies could help support students’ well-being.

## Data Availability

Data are available upon request. Requests to access these datasets should be directed to skyler.wyly@duke.edu.
