# Supplementary material for: Too little, too much: a 4-year longitudinal study of within-person deviations in propensity for overinvolvement and student well-being
Source: Front Psychol. 2026 Aug 25;17:1870777. doi: 10.3389/fpsyg.2026.1870777 (PMC13551328; doi:10.3389/fpsyg.2026.1870777)
Supplement: Supplementary file 1 [file Data_Sheet_1.pdf]

Supplementary Material

This supplement reports, for each random-intercept cross-lagged panel model (RI-CLPM), the full set of within-person autoregressive, cross-lagged, interaction, and quadratic estimates (posterior median, posterior SD, 95% credible interval, and two-tailed posterior *p*), together with model convergence information and within-person effect sizes (R^2). Three models are reported: a linear RI-CLPM (Model S1; the preregistered Hypothesis 1 specification, which yields a posterior predictive *p*-value), the preregistered moderation model that adds the within-person PFO × Resilience interaction (Model S2; source of the in-text Hypothesis 1 and Hypothesis 2 estimates), and the exploratory model that adds the within-person quadratic effect of PFO (Model S3).

Supplemental Table 1. Model convergence and fit Model Free param.

Model	Free parameters	Chains	Iterations	Final PSR	PPP
S1. Linear RI-CLPM (H1)	98	2	60,000	1.019	.37
S2. Moderation RI-CLPM (H1 & H2)	100	2	100,000	1.026	-
S3. Quadratic RI-CLPM (exploratory)	102	2	200,000	1.014	-

Note. PSR = Potential Scale Reduction factor (convergence criterion < 1.10). PPP = posterior predictive *p*-value. All models used Bayesian estimation (Mplus 8.7; Gibbs sampler, 2 chains, thinning = 2). Because the moderation and quadratic models contain latent product terms (XWITH) estimated under TYPE = RANDOM, Mplus does not compute a posterior predictive *p*-value, DIC, or conventional SEM fit indices for these models; PPP is therefore available only for the linear model (— = not available). Full PSR trajectories and complete output are available on OSF.

Effect-size computation. To convey the magnitude of effects, we report the unstandardized within-person estimates with 95% credible intervals (expressed in the outcomes' within-person units) together with the within-person R^2 for each outcome. We do not report fully standardized (STDYX) coefficients: Mplus does not produce a standardized solution for models containing latent product terms (XWITH) estimated under TYPE = RANDOM (our moderation and quadratic models), and standardized interaction and quadratic coefficients are difficult to interpret unambiguously. Because Mplus does not output model-implied latent variances for these models, withinperson R^2 was computed by the authors as 1 − (residual variance / model-implied total within-person variance), averaged across the predicted waves, where the total within-person variance at each wave was obtained by propagating the wave-1 within-person covariance matrix through the estimated autoregressive/cross-lagged coefficient matrix and adding the estimated residual (co)variances (a VAR(1) recursion consistent with the partialstationarity constraints; product-term variances under multivariate normality of the within-person factors). For the exploratory curvilinear effects, the

model-implied predicted curves (Figure 2) provide a more interpretable summary of magnitude than any single coefficient.

Supplemental Table 2. Linear Model: Within-Person Autoregressive and Cross-Lagged Effects among Propensity for Overinvolvement, Resilience, Sleep Quality, and Well-Being

Outcomes	Predictors	Estimate	*SD*	*p*-value	95% CI
**PFO**					
	$PW_{(t-1)}$	0.69	0.13	< .001	[0.43, 0.94]
	$RW_{(t-1)}$	0.09	0.11	0.368	[-0.13, 0.31]
	$SW_{(t-1)}$	-0.18	0.43	0.600	[-1.13, 0.61]
	$WW_{(t-1)}$	0.02	0.16	0.884	[-0.30, 0.34]
**Resilience**					
	$RW_{(t-1)}$	0.63	0.15	0.002	[0.33, 0.90]
	$PW_{(t-1)}$	0.08	0.07	0.264	[-0.07, 0.23]
	$SW_{(t-1)}$	-0.03	0.40	0.910	[-0.78, 0.89]
	$WW_{(t-1)}$	0.03	0.16	0.830	[-0.23, 0.40]
**Sleep Quality**					
	$SW_{(t-1)}$	-0.18	0.18	0.234	[-0.58, 0.12]
	$PW_{(t-1)}$	0.04	0.06	0.450	[-0.06, 0.16]
	$RW_{(t-1)}$	-0.04	0.09	0.640	[-0.20, 0.14]
	$WW_{(t-1)}$	-0.14	0.13	0.216	[-0.43, 0.07]
**Well-being**					
	$WW_{(t-1)}$	0.64	0.20	0.002	[0.24, 1.02]
	$PW_{(t-1)}$	0.00	0.07	0.952	[-0.14, 0.12]
	$RW_{(t-1)}$	-0.03	0.11	0.790	[-0.22, 0.23]
	$SW_{(t-1)}$	0.38	0.43	0.164	[-0.22, 1.55]

Note. Linear model includes only within-person autoregressive and cross-lagged paths (no latent product terms); it provides the posterior predictive *p*-value reported in Supplemental Table 1. Within-person terms are PFO (PW), Resilience (RW), Sleep Quality (SW), and Well-Being (WB); predictors are time lagged at $t-1$. Estimates are unstandardized path coefficients with standard deviations (*SD*); statistical significance is indicated with two-tailed *p*-values and 95% credible intervals (CIs).

Supplemental Table 3. Focused Model: Within-Person Effects of Propensity for Overinvolvement on Resilience, Sleep Quality, and Well-Being

Outcomes	Predictors	Estimate	*SD*	*p*-value	95% CI
**PFO**					
	$PW_{(t-1)}$	0.67	0.14	< .001	[0.42, 0.95]
	$RW_{(t-1)}$	0.09	0.12	0.432	[-0.16, 0.32]
	$SW_{(t-1)}$	-0.17	0.39	0.584	[-1.06, 0.53]
	$WW_{(t-1)}$	0.03	0.18	0.856	[-0.32, 0.39]
**Resilience**					
	$RW_{(t-1)}$	0.67	0.17	0.012	[0.29, 0.93]
	$PW_{(t-1)}$	0.08	0.08	0.276	[-0.07, 0.24]
	$SW_{(t-1)}$	-0.01	0.37	0.968	[-0.69, 0.84]
	$WW_{(t-1)}$	0.04	0.19	0.808	[-0.24, 0.48]
**Sleep Quality**					
	$SW_{(t-1)}$	-0.16	0.18	0.242	[-0.61, 0.13]
	$PW_{(t-1)}$	0.04	0.06	0.508	[-0.07, 0.15]
	$RW_{(t-1)}$	-0.04	0.11	0.668	[-0.26, 0.17]
	$WW_{(t-1)}$	-0.14	0.14	0.228	[-0.47, 0.08]
	$PWRW_{(t-1)}$	0.00	0.08	0.982	[-0.16, 0.15]
**Well-being**					
	$WW_{(t-1)}$	0.65	0.20	0.002	[0.25, 1.03]
	$PW_{(t-1)}$	0.01	0.07	0.906	[-0.12, 0.16]
	$RW_{(t-1)}$	-0.03	0.12	0.782	[-0.23, 0.26]
	$SW_{(t-1)}$	0.33	0.35	0.182	[-0.21, 1.21]
	$PWRW_{(t-1)}$	-0.09	0.10	0.316	[-0.32, 0.10]

Note. Focused model includes only the within-person interaction of interest (PFO × Res). Within-person terms are PFO (PW) on Resilience (RW), Sleep Quality (SW), and Well-Being (WB); predictors are time lagged at $t-1$. Estimates represents the unstandardized path coefficients, standard deviations (*SD*); statistical significance is indicated with two-tailed *p*-values and 95% credible intervals (CIs).

1 Propensity for Overinvolvement Confirmatory Factor Analysis

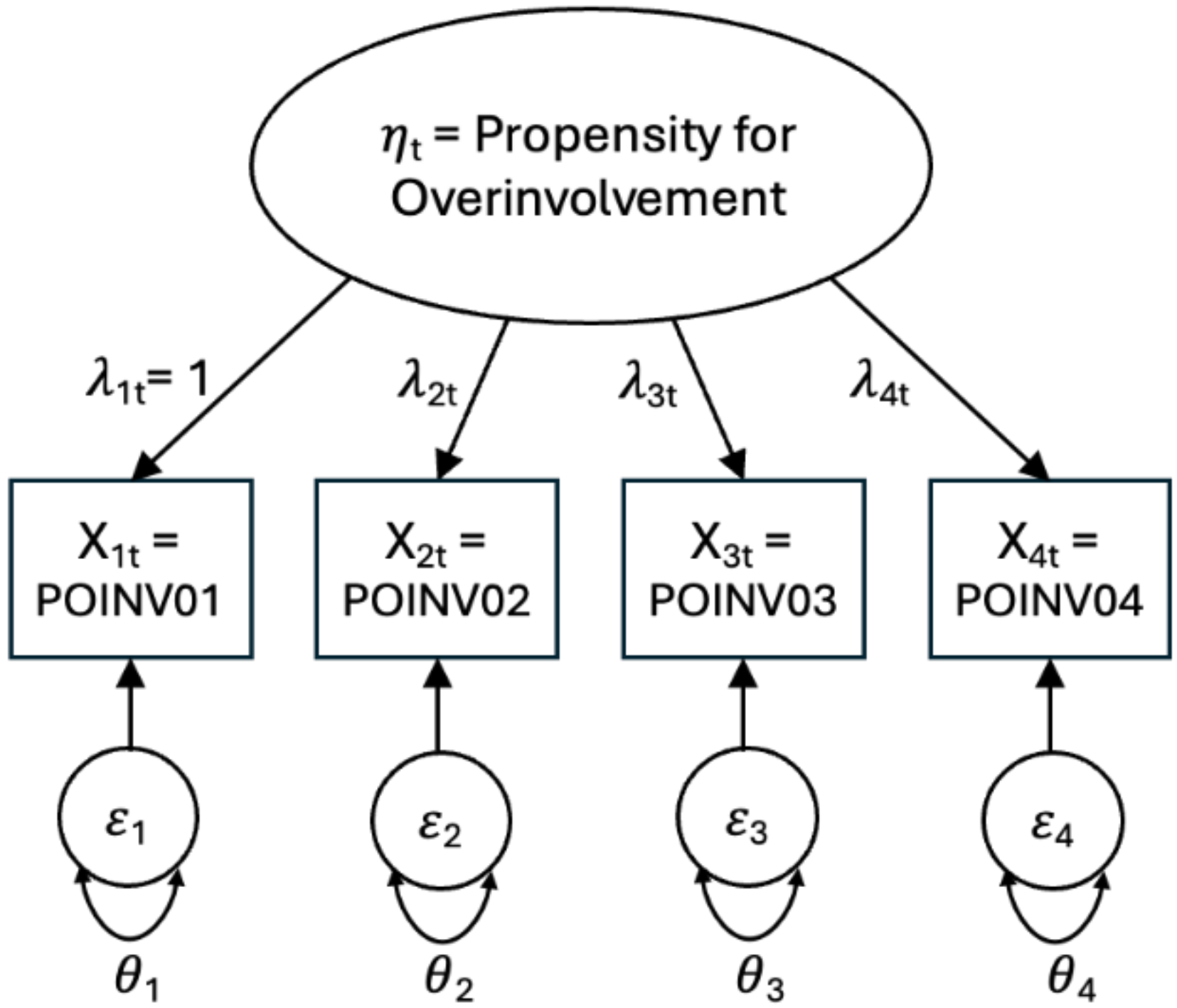

Supplemental Figure 1. Propensity for Overinvolvement measurement model.

Note: Measurement model for η_t, Propensity for overinvolvement at time t = 1-4. λ are factor loadings, ε are uniquenesses, and θ are residual error variances.

Supplemental Table 4

Propensity for Overinvolvement Confirmatory Factor Analysis Fit Indices and Reliability

Time	χ2	CFI	TLI	RMSEA	SRMR	Omega
First-year Fall	$\chi2(2) = 135.71$, $p < .001$	0.56	0.00	0.43	0.07	.80
Second-year Fall	$\chi2(2) = 49.61$, $p < .001$	0.82	0.45	0.28	0.09	.77
Third-year Fall	$\chi2(2) = 24.17$, $p < .001$	0.92	0.77	0.20	0.06	.81
Fourth-year Fall	$\chi2(2) = 72.85$, $p < .001$	0.75	0.24	0.36	0.09	.81

Note: Chi-square (χ2), comparative fit index (CFI), Tucker-Lewis fit index (TLI), root mean square error of approximation (RMSEA), and standardized root mean square residual (SRMR) fit indices for propensity for overinvolvement at each timepoint.

Supplemental Table 5

Propensity for Overinvolvement Two-Factor Confirmatory Factor Analysis Fit Indices

Time	χ2	CFI	TLI	RMSEA	SRMR	*r*
First-year Fall	$\chi^2(3) = 4.00$, p = .262	1.00	0.99	0.03	0.04	.64
Second-year Fall	$\chi^2(3) = 17.76$, p < .001	0.94	0.89	0.13	0.05	.57
Third-year Fall	$\chi^2(3) = 2.48$, p = .479	1.00	1.00	0.00	0.01	.73
Fourth-year Fall	$\chi^2(3) = 6.74$, p = .081	0.99	0.97	0.07	0.04	.64

Note: Two-factor CFA for PFO, with items 1 and 2 loading on an overinvolvement factor and items 3 and 4 loading on a FOMO factor. Chi-square (χ2), comparative fit index (CFI), Tucker-Lewis fit index (TLI), root mean square error of approximation (RMSEA), and standardized root mean square residual (SRMR), (*r*) standardized correlation between the two latent factors.

Supplemental Table 6. Sensitivity Analysis: Combined Model Including Both the PFO × Resilience Interaction and the PFO² Quadratic Terms

Effect (t−1 → t)	**Original model** b [95% CI], *p*	**Combined model** b [95% CI], *p*
PFO² → Resilience	−0.63 [−1.52, −0.16], .010	−1.40 [−5.04, −0.27], .004
PFO² → Sleep quality	1.22 [0.69, 2.90], < .001	1.87 [0.81, 5.54], < .001
PFO² → Well-being	−0.69 [−1.74, −0.27], .002	−0.90 [−3.54, −0.10], .034
PFO × Resilience → Sleep quality	0.00 [−0.16, 0.15], .982	0.71 [−0.73, 1.64], .412
PFO × Resilience → Well-being	−0.09 [−0.32, 0.10], .316	−1.08 [−2.62, 0.68], .378

Note. The "Original model" column reproduces the focal higher-order estimates from the separate models reported above—the PFO² quadratic effects from the quadratic model and the PFO × Resilience interaction effects from the focused model. The "Combined model" column reports the same effects from a single model that includes both sets of terms simultaneously (Propensity for Overinvolvement, Resilience, Sleep Quality, and Well-Being only; no covariates; 104 free parameters; 2 chains, 200,000 iterations; final PSR = 1.09). All three quadratic effects remained credibly different from zero and in the same direction, and both interaction effects remained non-significant, indicating that the curvilinear pattern is robust to inclusion of the moderation term. The wider credible intervals in the combined model reflect the added collinearity of estimating both second-order terms simultaneously. Estimates are unstandardized within-person path coefficients (posterior medians) with 95% credible intervals (CI) and two-tailed posterior *p*-values; all paths were constrained time-invariant (partial stationarity).
